# Exploring Challenges: A Qualitative Study on Occupational Therapy in Acute Psychiatric Inpatient Settings

**DOI:** 10.1155/oti/5521839

**Published:** 2026-06-16

**Authors:** Zahra Nobakht, Shokoufeh Ahmadi, Morvarid Ahadi, Ghazaleh Mandani, Mohsen Eslampanah

**Affiliations:** ^1^ Department of Occupational Therapy, School of Rehabilitation Sciences, Psychosis Research Center, University of Social Welfare and Rehabilitation Sciences, Tehran, Iran, uswr.ac.ir; ^2^ Health in Emergency and Disaster Research Center, University of Social Welfare and Rehabilitation Sciences, Tehran, Iran, uswr.ac.ir; ^3^ School of Behavioral Sciences and Mental Health, Psychosis Research Center, University of Social Welfare and Rehabilitation Sciences, Tehran, Iran, uswr.ac.ir; ^4^ Razi Educational and Therapeutic Center, Psychosis Research Center, University of Social Welfare and Rehabilitation Sciences, Tehran, Iran, uswr.ac.ir

**Keywords:** acute settings, inpatient care, mental disorders, mental health, occupational therapy

## Abstract

**Objective:**

Occupational therapy plays a crucial role in the rehabilitation and recovery of individuals facing acute psychiatric challenges. However, the practice within acute psychiatric inpatient settings is often fraught with unique challenges that can hinder effective intervention. This qualitative study is aimed at exploring the multifaceted obstacles and challenges that occupational therapists encounter in these environments.

**Method:**

This study utilized a qualitative approach with conventional content analysis. A total of 14 participants were involved, comprising active and experienced therapists, individuals currently hospitalized in acute settings, and psychiatrists. After obtaining ethical approval and informed consent, in‐depth, unstructured interviews were conducted in accordance with the research objectives. Purposeful sampling and maximum diversity strategies were employed to select and invite participants, who subsequently underwent in‐depth, semistructured interviews. The interviews were meticulously recorded and subsequently transcribed. The transcribed texts were subjected to analysis through the inductive qualitative content analysis.

**Results:**

The challenges associated with implementing occupational therapy interventions in acute psychiatric inpatient settings were categorized into five main areas: patient engagement, therapist safety concerns, burnout and high work pressure, lack of standard facilities and space, and unawareness of the need for such interventions.

**Discussion:**

Considering the various challenges faced by occupational therapists in acute psychiatric inpatient settings, a comprehensive and coordinated approach is essential. Issues such as patient engagement, therapist safety concerns, burnout and high work pressure, lack of standard facilities, and unawareness of the need for such interventions all impact the effectiveness of therapy. Ongoing training and education, coupled with supervision and mentorship, can empower therapists and promote self‐care practices to mitigate burnout. Fostering interdisciplinary collaboration, conducting regular meetings to discuss patient progress, and ensuring role clarity among team members are essential to facilitate comprehensive care. Providing adequate facilities and resources, ensuring safe working conditions, and raising awareness about the importance of occupational therapy are crucial.

## 1. Introduction

According to the World Health Organization (WHO), more than 500 million people worldwide experience mental health disorders, including nearly 10 million adults in the United States with severe mental illnesses [1] Nonetheless, even with the existence of evidence‐supported treatments, the rate of mental illness remains unchanged over time [2]. According to the WHO report, mental disorders are responsible for 15% of the years of life lost due to disease. These disorders mainly affect a person′s productive years in the personal and social spheres. In addition to individual aspects, this issue has a vital role in society from economic and social points of view. Mental illness–related disability can be crippling and may hinder or obstruct the growth of functional abilities related to personal hygiene, self‐care, self‐direction, social and interpersonal relationships, learning, and leisure activities [3]. Iranian people have encountered numerous stressful situations over the past several decades, such as war, significant economic sanctions, and various natural disasters in more recent times. These factors can increase the incidence of mental illness [4]. The report of the Social Insurance Organization of Iran has shown that the highest rate of disability is related to the Neuropsychological Commission, which alone accounts for 32% of all disabled people [5]. Whenever the patient′s symptoms are such that they pose a threat to him/her or the people around, admission to the inpatient ward is required [6]. It can improve therapeutic relationships and enhance patient involvement in recovery processes [7].

Occupational therapy interventions promote and foster active involvement in the recovery of psychiatric patients [8]. Despite the beneficial effects of occupational therapy and the critical role of occupational therapists as members of the psychiatric rehabilitation team in the recovery, the access of many of these patients to occupational therapy services is limited. [9]. Occupational therapists, as members of the psychiatric rehabilitation team, can significantly improve a person′s mental health, which includes better coping mechanisms for individuals dealing with chronic illnesses and supporting well‐being through encouraging involvement and participation in meaningful activities and occupation‐based interventions [10]. Therefore, occupational therapists are uniquely prepared to provide mental health rehabilitation services in an inpatient setting and when they are released from inpatient psychiatric care [11].

Some studies indicate that these patients have problems with their daily functions. After controlling their acute symptoms of the disease and reaching relative stability, they spend most of their time sleeping, watching TV, and eating. During hospitalization, patients perform the minimum physical and nonphysical targeted activity, which is approximately 17 min a day, and they perform nearly no physical or nonphysical activities for approximately 5 h a day [12]. Due to third‐level prevention and attempts to restore and preserve all or part of the patient′s lost abilities, occupational therapy groups and individual interventions are formed to increase patient performance and skills based on the patient′s diagnosis and symptoms during hospitalization.

Since the early 1980s, there has been a significant change in policy in most Western countries in terms of the treatment provided to people with mental illness. Once the inpatient setting was the focal point of care, it is now widely accepted that most care should be provided in the community setting [1]. This is evident in many critical mental health policy documents. However, acute mental health care is basic, intending to prompt psychiatric needs and stabilizing people encountering serious mental illness and requires continued development [13, 14]. The gradual decrease in the length of hospital stay of psychiatric inpatients has raised important questions about the role of occupational therapy in the acute mental health setting. Occupational therapists, concerned with improving daily functioning, are often frustrated by the lack of time to treat long‐term functional problems [15]. Considering the short‐term hospitalization, how should the occupational therapist′s role be better defined? What are the key challenges faced by occupational therapy in acute psychiatric inpatient settings? The physical environment, sociocultural environment, and pattern of activities of an inpatient psychiatric unit are not very similar to those of everyday life. Since the nature of each activity changes when separated from its natural context, hospitalized patients disconnect from familiar activities associated with it, which is accompanied by role dysfunction that often occurs with worsening symptoms. Therefore, dealing with issues related to daily functioning in the inpatient environment is a fundamental challenge for occupational therapists.

Duffy and Nolan discussed the changing nature of acute mental health and stated that if occupational therapists want to continue as one of the leading professional groups in the mental health field, the implications of recent and ongoing changes in mental health care should be considered. Occupational therapists must reflect on current practices, focus on clinical assessment, and debate appropriate evidence‐based interventions for the acute mental health setting [16].

Today, along with the journey of occupational therapy toward new horizons, the challenge of paying attention to the context appears to be the primary and unavoidable concern at the world level. People′s performance is always affected by the context in which it is performed [17]. Considering the undeniable differences in the implementation contexts and facilities between Western countries and those in the Eastern hemisphere, where existing occupational therapy solutions and models have been established and developed, we are aimed at identifying the challenges faced by occupational therapy in acute psychiatric inpatient settings. This understanding will help in preparing tailored occupational therapy guidelines for acute mental health settings.

## 2. Method

### 2.1. Study Design

This study employed a qualitative descriptive design using conventional content analysis [18]. Qualitative content analysis is a research methodology aimed at obtaining a succinct yet comprehensive description of the phenomenon, resulting in categories or concepts that encapsulate its essence [18]. In accordance with the principles of this approach, data were gathered directly from participants without any preconceived notions. The primary advantages of this method lie in its capacity to reflect the participants′ experiences, yielding a comprehensive portrayal of the phenomenon grounded in the subjective significance of those experiences [19, 20].

According to the Declaration of Helsinki, this study has been approved by the Ethical Committee of the University of Social Welfare and Rehabilitation Sciences (IR.USWR.REC.1400.289). The informed written consent was obtained from participants. They could leave the study at any time. Recording interviews were conducted only with the consent of the participants. This study was reported in accordance with the Standards for Reporting Qualitative Research (SRQR) checklist [21].

### 2.2. Participants and Sample

Purposeful sampling was employed in this study to capture diverse perspectives on the challenges of occupational therapy in acute psychiatric inpatient settings. [18]. Patients admitted to the acute mental health wards were selected considering variation in age, gender, marital status, and education level via purposeful contacts with ward heads. Therapists were chosen based on their educational background, years of work experience, and workplace context. In total, 14 participants were recruited: eight occupational therapists with 5–30 years of professional experience, five patients hospitalized in an acute psychiatric ward in Tehran (Iran) who were clinically stable enough to follow verbal instructions, and one psychiatrist working in the same hospital. Among the participants, 10 were women and 4 were men. Their ages ranged from the early 20s to above 50 years, with educational levels spanning from diploma to doctoral degrees. Marital status also varied, including both married and single participants (Table 1). All participants were recruited from the Razi Educational and Therapeutic Psychiatric Center, the largest psychiatric hospital in Iran public tertiary hospital with high caseloads (~500 beds). Recruitment continued until data saturation was achieved, meaning no new themes or codes emerged from subsequent interviews. All participants remained in the study and none were excluded. The number of participants was established in accordance with the principles of saturation. After doing 12 interviews, we had sufficient data and did not learn anything new from the interviews. After that, two more interviews were done to ensure beyond any doubt there was sufficient data. In line with qualitative research principles, participant selection is determined throughout the study, with purposeful sampling persisting until the researchers achieve saturation in any given concept, at which point the collection of additional data no longer contributes new codes or concepts. The psychiatrist′s data informed triangulation but was not quoted separately, as it corroborated existing themes.

**Table 1:** Profile of participated older adults.

Demographic characteristics		Number (percentage)
Age at time of intervwardiew	20–30 years old	2 (14.3%)
	30–40 years old	5 (35/7%)
	40–50 years old	4 (28/6%)
	50 years old and over	3 (21.4%)

Sex	Female	10 (71.4%)
	Male	4 (28/6%)

Marital status	Married	9 (64/2%)
	Widow	5 (35/7%)

Education	Diploma	3 (21.4%)
	Bachelor	6 (42.8%)
	Masters	3 (21.4%)
	Doctorate	2 (14.3%)

The amount of work experience of therapists	5–10 years	2 (22.2%)
	10–20 years	3 (33.3%)
	20–30 years	4 (44.4%)

### 2.3. Data Collection

This study first used open, semistructured face‐to‐face and individual interviews to collect data. Semistructured interviews make it conceivable to assess the participant′s perceptions and attitudes more profoundly. The interviews were conducted in Tehran from May 2023 to June 2024. Before starting each interview, while introducing herself and stating the purpose of the research, the researcher provided an informed consent document to the participants. After obtaining the consent form, the interview was conducted.

The interviews lasted between 21 and 75 min, with an average duration of 42 min. The location for the interviews was chosen by mutual agreement between the researcher and the participants, and they were conducted at the therapists′ workplaces for convenience. In addition to the interview data, the researchers collected and analyzed field notes, which served as complementary data to enhance trustworthiness. The commencement of each interview involved gathering demographic information, which was subsequently followed by an open‐ended question. The first question included “ Would you please talk about your experience about occupational therapy interventions in this facility?” “ What obstacles have you faced in receiving/ providing occupational therapy interventions?” The researcher employed these questions to direct the research topic and further examined the participants′ responses by posing probing inquiries.

### 2.4. Data Analysis

The data analysis process was conducted in accordance with the methodology outlined by Graneheim and Lundman (2004), concurrently with data collection. All interviews, which were audio recorded, were transcribed verbatim and meticulously compared with the original files for accuracy. To enhance trustworthiness, every stage of data analysis was independently executed by the first and second authors. Initially, each interview was read multiple times by the researchers to achieve deep immersion and comprehensive familiarity with the data. In the subsequent phase, we examined the entire text while delineating meaning units. A meaning unit is defined as words, sentences, or paragraphs that encompass interrelated aspects through their content and context. This process continued until all facets of the content pertinent to the research question were thoroughly described. We then coded the meaning units with appropriate labels and organized them into coding sheets. In the following step, through constant comparison and by considering the similarities and differences in the properties and dimensions of the explored concepts, we grouped analogous cases into coding sheets, leading to the development of subcategories and categories. Efforts were made to create categories that are internally homogeneous yet externally heterogeneous, comprehensive, and distinctive, thereby providing a holistic representation of the text′s content. Subsequently, the explored concepts were translated into English. The MAXQDA 2020 software was utilized to organize and analyze data frequently.

### 2.5. Rigor

The findings from content analysis studies must be reliable, and the methods employed to achieve these results should be critically assessed. In this study, to ensure the credibility of the data several approaches were utilized as follows:

• Prolonged engagement: The researchers engaged extensively with data collection and the research process and it lasted about 13 months, allowing them to gain insights into the participants′ culture and living conditions.
• Peer review: The researchers invited an expert, who was simultaneously involved in a qualitative project, to analyze certain segments of the data for peer validation. The analysis revealed no significant discrepancies between this peer review and the researchers′ own findings.
• Member checking: The data and their interpretations were verified and confirmed by the participants themselves (member check). Eight participants were member checked: five therapists, one psychiatrist, and two patients.

## 3. Results

This study is aimed at exploring the challenges of occupational therapy interventions in hospitalized patients of the acute mental health setting. According to the participants′ experiences, they were classified into five main categories, including patient engagement, therapist safety concern, burnout and high work pressure, lack of standard facilities and space, and unawareness of the need for interventions (Table 2).

**Table 2:** Challenges of occupational therapy interventions in hospitalized patients of the acute mental health setting.

Main	Subcategories	Primary
Patient engagement	Failure to provide internal and external incentives for participation	Activity mismatch with interest
		Activity mismatch with capability
		No reinforcement
	Remaining symptoms of the disease	Patient′s mood and behavioral changes
		Changeable and different symptoms
		Disruption of primary relationships
		The talkativeness and loudness of the patient
	Drug side effects	Drowsiness of the patient
		Patient′s lack of motivation

Therapist safety concern	Performing tasks unexpected by the patient	Performing risky tasks
		Conflicts in the ward
	Preventing unexpected tasks	Limiting the patient′s access
		The need for constant attention of the therapist

Burnout and high work pressure	The disproportion between the number of occupational therapists and the number of patients	Lack of occupational therapy staff
		Noncontinuity of 24‐h service
		Judicial patient and companion in the acute ward
	Inconsistency of patients	Presence of all kinds of patients in the acute department
		Not screening patients
		Act according to taste
	The lack of a coherent program	The occupational therapy program is not routine
		Not introducing occupational therapy according to routine
	The disruption of the continuity of intervention and rehospitalizations	No follow‐up after discharge
		The impossibility of family participation in the treatment process
		Being the only therapist in the ward

Lack of standard facilities and space	Lack of facilities and equipment and nonstandard physical space	Failure and lack of equipment
		Equipment inappropriateness
		Space limitation
		Space inappropriateness

Unawareness of the need for interventions	The lack of priority of occupational therapy services	Not introducing occupational therapy upon arrival
		Not consulting the occupational therapist
		The interference of programs within the ward with occupational therapy
	Limitation of referral	Taking case referrals for clients
		Refusal to refer
	Lack of knowledge and need for training, and changing the attitude of residents and colleagues to settings occupational therapy	Colleagues not knowing about occupational therapy services
		Residents not knowing about occupational therapy services
	Decreasing interest and motivation of the therapist	Decreased motivation due to insufficient knowledge of others
		Decreased motivation due to lack of equality with other personnel
	Lack of presentation of services	The limitation of the therapist′s ability to participate in the visit
		The novice therapist′s lack of experience

*Note:* Achieving saturation: Saturation refers to the repeated occurrence of data and the confirmation of extracted findings. In qualitative research, saturation is achieved when data repetition occurs, making it difficult to generate new findings or develop new concepts. This serves as a robust criterion for ensuring saturation. However, some qualitative researchers argue that saturation can only be achieved within a specific culture or phenomenon at a particular time. They suggest that interviewing and observing a different group may yield new findings from their unique experiences [19]. Speziale, H. S., Streubert, H. J. and Carpenter, D. R. 2011. *Qualitative Research in Nursing: Advancing the Humanistic Imperative*, Lippincott Williams and Wilkins.

## 4. Patient Engagement

Patient engagement consists of three subcategories: failure to provide internal and external incentives for participation, remaining symptoms of the disease, and drug side effects.

### 4.1. Failure to Provide Internal and External Incentives for Participation

Based on the participants′ statements, one of the challenges of occupational therapy interventions in the acute sector is the mismatch between activities and the interests and abilities of the patient. The following highlights the views of the participants.

Participant Number I2: “I did not go to occupational therapy because I do not know how to perform the activities.; if I do not know, why should I take someone else′s place?”

Participant Number T6: “There is another one, level of activities; for example, we had a patient who was a final year dental student…, the level of activity is really too basic and very simple for them, and we have to accept that person only in a group format, or if it is individual, they should tell us the activities they like, and we will provide activities within her/his capabilities and the resources we have, not general activities that are for all patients.”

### 4.2. Remaining Symptoms of the Disease

Based on the participants′ statements, one of the challenges of occupational therapy interventions in the acute sector is the patients′ mood and behavioral changes, as well as the variability and differences in symptoms. The following highlights the views of the participants.

Participant Number T6: “A patient who just came yesterday is still not stable; they still have suicidal thoughts, still experiencing their psychoses, and then you see them exhibiting aggressive behavior, showing suicidal behavior, and the occupational therapist is left wondering what to do. No matter how skilled they are, there are still impulsive behaviors from the patient that are beyond the control of both the psychiatrist, the occupational therapist, and the nurse.”

Participant Number T1: “One reason for this is that you cannot homogenize the patients together, and they are all in an acute phase. That is, someone who has depression is in an acute ward, someone who is manic is also in an acute ward, and schizophrenia is still uncontrolled, making it difficult to group them.”

### 4.3. Drug Side Effects

Based on the participants′ statements, one of the challenges of occupational therapy interventions in the acute sector is the patients′ drowsiness and lack of motivation. The following highlights the views of the participants.

Participant Number I1: “Unfortunately, they mostly give sleeping pills or sedatives, and we end up sleeping.”

Participant Number I1: “ it is a shame; after breakfast, we sleep for nearly two to three and a half hours at least, until snack time. This time is wasted; it is a pity for the patients to be here and not engage in their treatment. A general issue I see with the whole system here is that they make the patients sleep more. In sleep, a person does not grow; a person can solve their problems only when they can think, interact with others, and reach solutions, not when the problems are just there.”

## 5. Therapist Safety Concern

Therapist safety concern consists of two subcategories: performing tasks unexpected by the patient and preventing unexpected tasks.

### 5.1. Performing Tasks Unexpected by the Patient

Based on the participants′ statements, one of the challenges of occupational therapy interventions in the acute sector is the patient′s engagement in risky behaviors. The following highlights the views of the participants.

Participant Number T1: “A patient sitting next to you picks up a glue stick from the table and puts it in their ear—something unpredictable. It happened to me; I never expected them to do something like that. They took the glue and put it all in their ear, almost tearing their eardrum, or they take a crayon and scribble on the wall. All of this takes energy from us.”

Creating disturbances in the ward and disrupting the section is another challenge for occupational therapy services as highlighted by the participants.

Participant Number T1: “They get involved with the nursing staff; in terms of communication, some really have a viscosity that affects the station. They do not make accurate judgments, do not accept the time, and do not follow the ward rules. They get involved over a single cigarette. Their communication problems are significant.”

### 5.2. Preventing Unexpected Tasks

Regarding the previous challenges, the occupational therapist inevitably imposes restrictions in the ward, which itself presents a challenge in providing occupational therapy services in the acute sector. The following highlights the views of the participants.

Participant Number T3: “Not having a patient who is suitable for occupational therapy right now, coming in and disrupting the room, or the risks involved—if all my patients were stable, I would definitely have a different performance. Maybe I would even let them be free, perhaps I would provide them with materials, but in this ward, all my energy goes into preventing the patient from doing something.”

Participant Number I3: “I was waiting for it to open so we could go in and write something, without the fear of a nurse coming in and taking you. I would also beg and plead to get paper from anywhere. But your question was about how we got acquainted… I was waiting for it to open, and when it did, I went in and started writing comfortably, not like a hidden criminal.”

Participant Number I1: “Here, it seems like there is a hostility towards people, and on the pretext that people commit suicide, they say they will not provide you with paper because you will commit suicide. If your real concern is that, then at least provide some scrap paper. If the worry is that I might use my scarf to harm myself, well, those who commit suicide are different from someone who might use a razor….”

All the above challenges require the therapist to constantly check all patient behaviors and materials, necessitating the therapist′s continuous attentiveness. The following highlights the views of the participants.

## 6. Burnout and High Work Pressure

Burnout and high work pressure concern consists of four subcategories: the disproportion between the number of occupational therapists, the number of patients, inconsistency of patients, the lack of a coherent program, the disruption of the continuity of intervention and rehospitalizations.

### 6.1. The Disproportion Between the Number of Occupational Therapists and the Number of Patients

According to the participants′ statements, one of the challenges of occupational therapy interventions in the acute sector is the insufficient occupational therapy staff and the lack of continuous 24‐h services. The following highlights the views of the participants.

Participant Number T9: “In the morning shift, patients may be more involved with visits from psychologists, psychiatrists, and residents, and it can be said that the boredom, impatience, and restlessness of patients increases from the afternoon shift until bedtime. For example, consider that patients are extremely unstructured and idle for about 4–5 h in the afternoon. If we can keep occupational therapy active in the hospital both in the morning and in the afternoon, and of course with efficient staff and sufficient numbers, it would significantly help alleviate the patients′ boredom, impatience, and the fact that they really do not know what to do.”

Participant Number T4: “Many times, patients really have no program, and they start interacting with each other. If they are delusional or paranoid, this problem worsens. Some patients have personality issues, which is why they have behavioral and personality problems. When they are idle and unstructured, their conflicts with each other increase.”

### 6.2. Inconsistency of Patients

According to the participants′ statements, one of the challenges of occupational therapy interventions in the acute sector of judicial patients and their companions is the presence of all types of clients in the acute sector and the lack of patient screening. The following highlights the views of the participants.

Participant Number I4: “I have told the staff here many times that you have all kinds of cases here. Most of the cases are related to domestic violence. For example, in the U.S., these are separated; the domestic violence cases I have specifically researched are distinct from psychological cases. If someone is a prisoner, they are also separate. Here, even the judge… there is a woman who went to the presidency to protest certain issues and was brought here. In other words, all kinds of people are mixed together here.”

Participant Number T2: “These are level determinations. Now, in the hospital, in the ward, we have all kinds of patients. Classifying these patients based on a standard list is necessary, so that we can measure them quantitatively and categorize them, allowing us to say what we expect from this patient at this level.”

### 6.3. The Lack of a Coherent Program

Based on the participants′ statements, one of the challenges of occupational therapy interventions in the acute sector is the subjective nature of practice, the lack of routine in occupational therapy programs, and not introducing occupational therapy according to standard procedures. The perspectives of the participants are mentioned below.

Participant Number T6: “That depends on the occupational therapist; they choose which visit to attend, it is not mandatory, and there is no imperative from psychiatrists that you must be in my visit, it depends on preference.”

Participant Number T2: “Other therapists do have routines because they also have their own standards. Psychologists have routines; each psychologist selects a doctor, takes a history, and provides consultation. But our work is not routine‐based at all; I might take a patient into the room one day and close the door, or I might take 10 people one day, or I might even walk with a patient in the hallway or sit next to their bed. There is absolutely no routine, and this lack of order does not provide an interesting perspective from the viewpoint of others.”

### 6.4. The Disruption of the Continuity of Intervention and Rehospitalizations

Based on the participants′ statements, one of the challenges of occupational therapy interventions in the acute sector is the lack of follow‐up after discharge, the inability of family members to participate in the treatment process, and the isolation of the therapist in the department. The perspectives of the participants are mentioned below.

Participant Number T3: “No, it should not be cut off; we cannot follow up, we cannot pursue it. We can only provide consultation, but it is unclear if it will be effective. Does the patient have the mental capacity to follow our advice? Will their family even allow it? Sometimes the home environment is stressful; a 40‐m^2^, one‐bedroom apartment with four people, everyone is involved, and whoever is there feels bad. There should be a space where they can come to work and have some income.”

Participant Number I1: “About three nights ago, they came here. From the first night, they were screaming, and when I saw what their problems were, I asked, ‘What would you do if you had my problems?’ If people knew that… But I saw on the other side how easily everyone expresses their problems, and then the psychologist does not stigmatize the person. They say, ‘Your issues are this and that,’ and then they also mention the person′s difficulties, stating that they are struggling with such psychological issues at home. Because nothing is one‐sided, the family is a crucial part of the situation, and that has been ignored. The focus is on the patient, and with the notion that they are either mentally ill or crazy, it seems as if a prescription has already been written for them, and the matter is concluded.”

## 7. Lack of Standard Facilities and Space

Lack of standard facilities and space consists of two subcategories: lack of facilities and equipment and nonstandard physical space.

### 7.1. Lack of Facilities and Equipment

According to the participants, one of the challenges of occupational therapy interventions in the acute section is the lack and damage of equipment and the unsuitability of the tools. The participants′ viewpoints are mentioned below.

Participant Number T1“For example, we had a dart that was broken for a long time, and we did not have any darts. We recently got another one.”

Participant Number I4: “Sitting on these chairs is hard. Imagine you have to sit for 3 h to do a calming and recreational activity; these are unsuitable chairs, and we do not have anything like a lobby for patients to sit together and chat.”

### 7.2. Nonstandard Physical Space

According to the participants, one of the challenges of occupational therapy interventions in the acute section is the limited space and the unsuitability of the environment. The viewpoints of the participants are mentioned below.

Participant Number T3: “I have to keep calling them and coming up, calling them and coming up. This creates a problem; for example, if 20 people come down, and another 20 come up, they will not fit in the room. This is an issue we have in this section.”

Participant Number T2: “There should be a standard space; whatever is defined, we are not making anything up. We follow what the higher authorities say, like the Medical Council and the Treatment Department of the University, which we are under. If you want to set up a psychiatric occupational therapy clinic here, they give you guidelines that you must follow, from the area to the door, window, water, sink, it is all there.”

## 8. Unawareness of the Need for Interventions

Unawareness of the need for interventions consists of five subcategories: the lack of priority of occupational therapy services, limitation of referral, lack of knowledge and need for training, changing the attitude of residents and colleagues to settings occupational therapy, decreasing interest and motivation of the therapist, and lack of presentation of services.

### 8.1. The Lack of Priority of Occupational Therapy Services

The overlap of departmental programs with occupational therapy is another challenge for occupational therapy services in the acute section. The viewpoints of the participants are mentioned below.

Participant Number T5: “Work starts at 8:30, and some of our activities overlap with the nursing department. For example, at 8:30, not many patients come in, or if they do, they keep going out because it is their blood pressure time, and at 10, it is their break time.”

Participant Number T7: “Many times, the department does not cooperate. For instance, we are holding a group session, and suddenly the doctor comes in wanting to see a patient and leave right away. This disrupts everything; there are some problems like this.”

### 8.2. Limitation of Referral

One of the significant challenges that occupational therapists face as service providers is related to referrals. Obtaining case referrals and refusal to refer are challenges that therapists encounter daily. The viewpoints of the participants are mentioned below.

Participant Number T6: “I take a look at the file to see if it is appropriate or not. If it is suitable, I go to the doctor and ask for a referral for them.”

Participant Number T2: “The specialist doctor has sent a letter to the treatment department, which referred it to me, saying that I will not write a referral for occupational therapy for the patient to avoid imposing costs on the patient′s family. However, they need to come and use the service, but I will not make this an official referral because of the costs for their family.”

### 8.3. Lack of Knowledge and Need for Training, and Changing the Attitude of Residents and Colleagues to Setting Occupational Therapy

The lack of knowledge and the need for training and a change in the attitudes of colleagues and residents is a significant challenge that occupational therapists in the acute section face. The viewpoints of the participants are mentioned below.

Participant Number T3: “Why? First of all, we do not know each other, meaning the nurse, the social worker… For example, a few days ago, I was listening and they were saying they saw a picture of occupational therapy with someone in a wheelchair and another person giving a massage. Well, what does that have to do with occupational therapy? Occupational therapy is not about those things; it is about painting and so on. There is no interdisciplinary perspective regarding different fields; this is a problem. Or for example, one of my colleagues called me that day saying patients here complained that you did not play the song we liked. Well?? I know that person very well; they are a very cooperative person, very supportive. There is absolutely nothing wrong in this regard, and they really want teamwork, but they do not know what occupational therapy is… This is a problem; I think it is the biggest issue among the staff, and the newcomers are trying to reduce this.”

Participant Number T6: “Another challenge we have is that some of our psychiatrists still do not accept occupational therapy as a therapy or treatment. Now, go there, and they might give you a few puzzles to do, while the occupational therapist is dedicating time. Many symptoms that may not be identified by the assistant, social worker, psychologist, or even the psychiatrist themselves can be uncovered by the occupational therapist in 45 min.”

### 8.4. Decreasing Interest and Motivation of the Therapist

The decrease in motivation due to insufficient recognition from others and lack of equality with peers is a significant challenge that occupational therapists face as service providers. The viewpoints of the participants are mentioned below.

Participant Number T2: “If they do it, those are the good ones who call one by one. I want to mention that the motivational factor is not solely financial; in my opinion, it might come last. If you achieve job satisfaction and get feedback on your personality from your job, it ends up at the bottom. Financial feedback, that is my opinion. This does not happen in this hospital for the staff, especially our staff. I believe that after the doctors here, in terms of academic grade and employment grade, we in rehabilitation—physiotherapy and occupational therapy—are next after doctors. You see the universities where the staff studied. Once, in a meeting, the policy stated that a psychologist must be present for walking sessions. My colleague went to the psychologist and said, ‘Let us go for a walk.’ The psychologist told him, ‘You are not coming here anymore; I have a PhD in [subject],′ I will not name it, it is still there. In the first meeting I attended, I said, ′Mr. [name], you know better than I do; you are in academic systems, you know. My colleague got his degree from one of the best universities in the country, and this caused a lot of noise. Why did you say that? Your comment is inappropriate.’”.

Participant Number T2: “The work reaches a point where… there is something we have in management systems, a motivational system. Now, if I look for the book, it is there, called the principle of expectancy and equity in organizations. When you do not feel equality with your peers in terms of education and work experience, and your expectations are not met, employee motivation decreases. This is a law that we did not make up; it is based on motivational management theories. Staff come to my room; this is the occupational therapy unit. Occupational therapy management starts here. How many occupational therapists do you see here? Just me. In the management system of occupational therapy, it is only me.”

### 8.5. Lack of Presentation of Services

The limitation of the therapist′s ability to participate in visits and the lack of skills of novice therapists are challenges that occupational therapists face as service providers. The viewpoints of the participants are mentioned below.

Participant Number T4: “It somewhat depends on the therapist′s confidence and assertiveness. Usually, the more novice therapists have a fear of being part of the team during visits, thinking about what to say and how to say it. But I have always been there and have always addressed issues assertively, which has improved my relationship with the doctor and the patient. It has been beneficial for the patient because we are together there; I might mention a symptom, and they might have a comment as well. These visit sessions have really helped my learning; many times, the terms mentioned that I have not heard before, I easily ask the doctor what they are, and they explain them to me. In fact, it has been a learning session as well.”

Participant Number T1: “I have work, but for teamwork to take shape, the doctor has a significant effect on how they treat you or, for example, ask you… In the visit session, until they ask, it is a bit heavy for someone to speak… ‘Mr. Doctor, for example, I think the patient is a bit difficult; it is hard for me. Maybe if I were more assertive.”

## 9. Discussion

On the first day of stay in the acute mental health setting, due to the unpredictability of behavior, tolerance and unstable symptoms, especially in the first 2 days, as a general guideline, it is advisable to conduct brief one‐on‐one sessions lasting 20 min or less, particularly during the initial days. The frequency of hospitalization is typically determined based on the recommendations of the team [17]. The number of therapy sessions in the studies was usually 3–5days per week, and each session lasted approximately 45 min–2 h.

This study′s qualitative descriptive design, drawing from diverse perspectives at Iran′s largest psychiatric facility, uncovered five challenge categories: patient engagement, therapist safety concerns, burnout and high work pressure, lack of standard facilities and space, and unawareness of intervention needs. These reflect broader challenges in acute OT practice, including role constraints and workplace limitations, while also drawing attention to the distinct contextual nuances of under‐resourced settings [22]. The challenges of nurses working in the psychiatric setting in Ahmadi and Eskandari′s study in 2018 were divided into five categories, which are neglecting the psychiatric department and turning it into a place of exile, lack of standard conditions in the ward, increased risk of physical, mental, and social injuries, weakening of work motivation, and failure to meet financial and road expectations [23].

Persistent symptoms, medication side effects (e.g., drowsiness diminishing motivation), and mismatches between activities and patients′ interests or abilities emerged as central barriers to engagement. These factors often led to minimal participation, despite OT′s potential to promote meaningful occupations. This interpretation highlights how the volatility of the acute phase undermines the therapeutic alliance, reinforcing cycles of inactivity that worsen functional decline—aligned studies reported patients averaging only 17 min of purposeful activity per day. Such challenges may even arise in the early days of hospitalization following medication adjustments. Even when conditions appear favorable and patients enter the OT room, lack of motivation often persists if activities fail to align with their interests and abilities. Supporting our findings, recent Australian guidelines for inpatient OT engagement emphasize the importance of person‐centered, incentive‐based strategies to counteract symptom‐related disinterest, showing up to 25% improved adherence in graded activity pilots [24]. Iranian settings demand approaches blending low‐cost, culturally resonant activities (e.g., community‐inspired crafts).

Unexpected patient behaviors—such as impulsive misuse of materials or ward disruptions—demanded continuous vigilance and activity restrictions, shifting OT spaces from enabling environments to surveilled ones. This dynamic helps explain the emotional strain of risk anticipation, as therapists invested disproportionate energy in prevention rather than facilitation, mirroring the “safety funnel” of psychiatric units where control often overrides therapeutic freedom. In a study to explain patients′ experience of safety in the inpatient department of psychiatry, it has been pointed out that the participants′ narratives were centered around the axes of help, protection, and power. This study presents findings related to safety experience. Participants expected to be safe from themselves and others. At first, they felt secure from the outside world. Lack of awareness of copatients made them feel vulnerable. Participants expected nurses to keep them safe and felt safer when male nurses were present. Our findings align with 2024 investigations of OT in short‐stay units, which noted that tool‐related hazards increased patient vulnerability but simultaneously positioned therapists as de‐escalation specialists; notably, 68% of OTs reported that adaptive protocols effectively reduced incidents [25].

Another challenge faced by the occupational therapist is the nonpriority of occupational therapy services; for example, if the patient is doing activities in the occupational therapy department and at the same time the doctor has come to visit the patient, the patient is called for a visit. The same applies to nursing routines. Sometimes, these cases are the cause of lack of knowledge or attitude of psychiatrist or colleagues toward occupational therapy services and its importance. It has been reported that service users and health professionals often misunderstand the role of occupational therapy. In a study examining the relationship between occupational therapists and other team members, they found corroborating evidence for this. Their findings showed that although occupational therapists are appreciated, the amount of value their role can add is often not recognized [26, 27]. It has been claimed that occupational therapy is often mainly related to relieving boredom in the department [28]. One of the key challenges in this study is the lack of knowledge and the need for training regarding occupational therapy. Many residents and colleagues may not fully understand the significance and benefits of occupational therapy and relating occupational therapy to relieving boredom, which can lead to negative attitudes or indifference toward this treatment approach. Changing the attitudes of residents and colleagues toward occupational therapy can facilitate better acceptance of this therapeutic method and enhance collaboration in the treatment process.

Another challenge for occupational therapists is related to the environment and space of the occupational therapy department, which is similar to other inpatient departments. The advantages of a structured and organized environment in mental health settings have been identified in occupational therapy. An orderly and attractive unit is essential for proper mental health and in raising the morale of both the patient and the therapist. All supplies and equipment should have a designated storage area when not in use, and it is the therapist′s responsibility to ensure that each item is returned to its proper place or just left in a corner or piled on a shelf. In addition, the work environment should not be boring and depressing. Through innovation and ingenuity, any room can be made attractive and exciting with inexpensive materials and painting, and many patients enjoy working with them and are proud of the end result. Sometimes, environmental changes can be straightforward, but with careful design and appropriate equipment, a safe and comfortable space for pursuing recovery goals can be created. The occupational therapy room should be welcoming, appealing, and secure. Also, the equipment and facilities that exist in this department should be appropriate to the goals of occupational to increase self‐efficacy, responsibility, and socialization; for example, sometimes the occupational therapist may ask the person to activities may include feeding the fish in the aquarium, watering the potted plants, preparing tea, assisting in gathering and organizing the supplies needed for the activity, and cleaning the tables.

## 10. Conclusion

The findings of this study underscore the critical role of occupational therapy in acute psychiatric inpatient settings, highlighting both the significant benefits it can provide and the multifaceted challenges that therapists face. The challenges identified—ranging from issues of patient engagement and therapist safety to burnout and inadequate facilities—illustrate the need for a comprehensive approach to enhance the effectiveness of occupational therapy interventions.

To address these challenges, it is essential to foster an environment that prioritizes mental health rehabilitation through ongoing education, interdisciplinary collaboration, and adequate resource allocation. By increasing awareness of the value of occupational therapy among healthcare teams and ensuring that therapists have the support and facilities they need, we can improve patient outcomes and promote a more effective therapeutic environment. It is recommended that policymakers and professionals support occupational therapy by facilitating appropriate education and training, raising public awareness, ensuring the availability of necessary resources and facilities, and implementing measures to enhance therapists′ motivations.

Ultimately, addressing these obstacles will not only empower occupational therapists but also enhance the overall care provided to individuals facing acute psychiatric challenges, thereby contributing to their recovery and reintegration into daily life.

## 11. Limitations

Although efforts were made to include different perspectives, the sample consisted primarily of experienced therapists and hospitalized patients, potentially overlooking insights from other stakeholders, such as family members or administrative staff. Another constraint is that the findings of this study are context‐specific and may not generalize to other cultural or institutional settings.

The challenges identified may be specific to the particular acute psychiatric settings studied and may not fully represent the experiences of occupational therapists in different cultural or institutional contexts.

We suggest the following approaches:

• Expand research to include a more diverse range of participants, including different cultural backgrounds, age groups, and types of mental health disorders, to better understand the unique challenges and needs of various populations.
• Explore comparative studies between acute psychiatric settings and other mental health environments (e.g., outpatient clinics and community settings) to identify best practices and transferable strategies for occupational therapy.
• Examine the impact of interdisciplinary collaboration on the delivery of occupational therapy services, focusing on how teamwork influences patient engagement and overall treatment effectiveness.

## 12. Implications for Practice

This study highlights several practice‐level considerations for improving the delivery of occupational therapy in acute psychiatric inpatient settings. Strengthening interdisciplinary collaboration through structured education on the role of occupational therapy may enhance role clarity and referral practices within multidisciplinary teams. Adequate staffing levels, such as a ratio of one occupational therapist per 10–15 inpatients, are necessary to support consistent service provision and reduce practitioner burnout. In addition, access to dedicated therapeutic spaces equipped with sensory modulation resources is essential to facilitate effective, engagement‐focused interventions and support inpatient recovery.

## Author Contributions

M.A. and G.M. reviewed the literature and conceived the study. Z.N. and S.A. were involved in protocol development, gaining ethical approval, patient recruitment, and data analysis. M.E. prepared the first draft of the manuscript. All authors reviewed and edited the manuscript.

## Funding

This study was supported by the University of Social Welfare and Rehabilitation Sciences (10.13039/501100008974) (IR.USWR.REC.1400.289).

## Disclosure

All authors approved the final version of the manuscript.

## Conflicts of Interest

The authors declare no conflicts of interest.

## Data Availability

The data that support the findings of this study are available on request from the corresponding author. The data are not publicly available due to privacy or ethical restrictions.
